# *In vitro* performance of lipid-PLGA hybrid nanoparticles as an antigen delivery system: lipid composition matters

**DOI:** 10.1186/1556-276X-9-434

**Published:** 2014-08-27

**Authors:** Yun Hu, Marion Ehrich, Kristel Fuhrman, Chenming Zhang

**Affiliations:** 1 Department of Biological Systems Engineering, Virginia Tech, Blacksburg, VA 24061, USA; 2 Department of Biomedical Sciences and Pathobiology, Virginia Tech, Blacksburg, VA 24061, USA; 3 Veterinary Medicine Experiment Station, Virginia Tech, Blacksburg, VA 24061, USA

**Keywords:** Hybrid NP, Lipid composition, PLGA, Surface charge, Vaccine, Antigen delivery

## Abstract

Due to the many beneficial properties combined from both poly(lactic-co-glycolic acid) (PLGA) nanoparticles (NPs) and liposomes, lipid-PLGA hybrid NPs have been intensively studied as cancer drug delivery systems, bio-imaging agent carriers, as well as antigen delivery vehicles. However, the impact of lipid composition on the performance of lipid-PLGA hybrid NPs as a delivery system has not been well investigated. In this study, the influence of lipid composition on the stability of the hybrid NPs and *in vitro* antigen release from NPs under different conditions was examined. The uptake of hybrid NPs with various surface charges by dendritic cells (DCs) was carefully studied. The results showed that PLGA NPs enveloped by a lipid shell with more positive surface charges could improve the stability of the hybrid NPs, enable better controlled release of antigens encapsulated in PLGA NPs, as well as enhance uptake of NPs by DC.

## Background

Nanoparticles made from poly(lactic-co-glycolic acid) (PLGA) or lipids have been used as drug delivery systems for many years. PLGA and liposome nanoparticles (NPs) share some common merits, such as long circulation time, biocompatibility, tunable size, and high drug loading capacity [1,2]. Meanwhile, both PLGA and liposome NPs have their own unique advantages. For example, the degradation rate of PLGA NPs can be flexibly controlled by adjusting the molar ratio between glycolic acid and lactic acid [3], and substances with distinct physiochemical properties, including proteins, anticancer drugs, nucleic acids, and even metal NPs, can be easily incorporated into PLGA NPs. On the other hand, liposome NPs can entrap hydrophobic drugs between lipid layers while encapsulating hydrophilic payloads in the aqueous core. In addition, the surface chemistry of liposomes can be easily tuned to meet different requirements by simply adjusting the types or concentrations of lipids, and the inclusion of certain lipid molecules with terminal reactive groups offers great flexibility in conjugating target molecules with different chemical properties [4]. It is even possible to formulate liposomes that are sensitive to a wide range of external stimuli, such as heat, light, ultrasound, and pH, to allow a highly controlled release of payloads [5]. However, PLGA and liposome NPs also have their own limitations. For instance, the fabrication process for liposomes of accurate size is cumbersome [6], and they are also plagued by storage instability and burst release of the payload [7]. PLGA NPs, on the other hand, tend to have a short half-life during circulation *in vivo*[7], and the surface chemistry of PLGA NPs cannot be easily modified. Therefore, it would be attractive to fabricate lipid-PLGA hybrid NPs, which combine the desirable characteristics of both liposome and PLGA NPs, meanwhile mitigating or even avoiding the aforementioned limitations.

Indeed, in the past decade, lipid-PLGA hybrid NPs have exhibited great potentials as a delivery system for cancer drugs, antigens, as well as *in vivo* imaging agents. They may play an important role in overcoming the increasingly prevalent multidrug resistance (MDR) [8]. Encapsulation of anticancer drugs in both the PLGA core and the lipid layer allows the release of drugs in a stepwise manner, resulting in improved therapeutic index with reduced toxicity [9]. In vaccine application, vaccines delivered by hybrid NPs demonstrated an enhanced immunogenicity [10]. Antigens can be either conjugated on the surface of the lipid layer, or encapsulated inside the PLGA core, or both. In addition, molecular adjuvants such as monophosphoryl lipid A (MPLA) and CpG oligodeoxynucleotides (CpG OND) can be co-delivered with antigens to further enhance immune response and reduce systemic toxicity [11].

Despite the broad applications of lipid-PLGA NPs, some fundamental questions have not been well addressed. Among them, the surface chemistry of the hybrid NPs that is governed by lipid composition and concentration, including surface charge, hydrophobicity, fluidity, permeability, and steric shielding effect of polyethylene glycol (PEG) [12], could greatly impact the performance of the NPs as a delivery vehicle. The understanding of how a lipid shell affects the efficacy of drug or antigen delivery may provide basis for a more rational design of hybrid NPs. Therefore, in this study, lipid-PLGA NPs, which are composed of a PLGA core and a lipid shell with variable lipid compositions, were prepared. In order to evaluate the performance of the hybrid NPs as an antigen delivery system, a model antigen, keyhole limpet hemocyanin (KLH), was enclosed inside the PLGA core. The influence of different lipid compositions on the surface charge, size, and stability of hybrid NPs was evaluated. Furthermore, the release of KLH from the hybrid NPs in phosphate-buffered saline (PBS), fetal bovine serum (FBS), and human serum was studied. The *in vitro* uptake of the hybrid NPs with different surface properties by dendritic cells (DCs) was also studied. It was found that lipid shells made from cationic lipids could improve the stability of NPs, enable more controlled release of antigen, and enhance the uptake of the NPs by DCs. These results should provide guidance to future design of hybrid NPs for improving drug or antigen delivery.

## Methods

### Materials

Lactel® 50:50 PLGA was purchased from DURECT Corporation (DURECT Corporation, Cupertino, CA, USA). Lipids, including 1,2-dioleoyl-3-trimethylammonium-propane (DOTAP), 1,2-dioleoyl-sn-glycero-3-phosphocholine (DOPC), 1,2-distearoyl-sn-glycero-3-phosphoethanolamine-*N*-[amino(polyethylene glycol)-2000] (ammonium salt) (DSPE-PEG2000), and 1,2-diphytanoyl-sn-glycero-3-phosphoethanolamine-*N*-(7-nitro-2-1,3-benzoxadiazol-4-yl) (ammonium salt) (NBD PE), were purchased from Avanti Polar Lipids, Inc. (Avanti Polar Lipids, Inc., Alabaster, AL, USA). KLH, poly(vinyl alcohol) (PVA; Mw 89,000 to 98,000), dichloromethane, rhodamine B, sodium deoxycholate (DOC), trichloroacetic acid (TCA), sodium dodecyl sulfate (SDS), paraformaldehyde, and Triton™ X-100 were purchased from Sigma-Aldrich Inc. (Sigma-Aldrich Inc., Saint Louis, MO, USA). 1-Ethyl-3-[3-dimethylaminopropyl] carbodiimide hydrochloride (EDC) was purchased from Thermo Fisher Scientific Inc. (Thermo Fisher Scientific Inc., Waltham, MA, USA). JAWSII (ATCC® CRL-11904™) immature DCs were purchased from ATCC (Manassas, VA, USA). FBS, granulocyte-macrophage colony-stimulating factor (GM-CSF) recombinant mouse protein, minimum essential medium (MEM) α, trypsin/ethylenediaminetetraacetic acid (EDTA), and HCS CellMask™ Blue Stain were purchased from Life Technologies Corporation (Life Technologies Corporation, Grand Island, NY, USA).

### Fabrication of PLGA-KLH (PK) nanocomplex

PLGA-KLH nanocomplex was prepared using double emulsion solvent evaporation method [13]. Briefly, PLGA of 200 mg was dissolved in 5 mL dichloromethane, followed by mixing with 300 μL of 10 mg/mL KLH using a vortex mixer for 2 min. The resulting mixture emulsified via sonication at 20% amplitude for 20 s using a sonic dismembrator (Model 500; Fisher Scientific, Pittsburgh, PA, USA). The primary emulsion was added dropwise into 200 mL 1% (*w*/*v*) PVA and stirred for 10 min at 500 rpm. The above suspension was emulsified through sonication at 50% amplitude for 120 s. The secondary emulsion was stirred overnight to allow organic solvent to evaporate. After settling at room temperature for 30 min, precipitant was removed. NPs in suspension were collected by centrifuge at 20,000 *g*, 4°C for 30 min (Beckman Coulter Avanti J-251, Brea, CA, USA). Pellet was washed using ultrapure water for three times. The final suspension was freeze dried (LABCONCO FreeZone 4.5, Kansas City, MO, USA) and stored at 2°C for later use.

### Assembly of liposome-PK (LPK) nanocomplex

Lipid film of 20 mg with various lipid compositions was hydrated with 15 mL hydration buffer (0.9% saline, 5% dextrose, and 10% sucrose). After vigorous mixing with vortex for 2 min, the resulting solution was incubated in a 55°C water bath for 5 min and cooled to room temperature. PK NPs of 200 mg were added into liposome solution and pre-homogenized for 15 min using Branson 2510 bath sonicator (Branson Ultrasonics Corporation, Danbury, CT, USA), followed by sonication in ice bath at 15% amplitude for 5 min (pulse on 20 s, pulse off 50 s) using a sonic dismembrator (Model 500; Fisher Scientific, Pittsburgh, PA). The formed LPK NPs were collected by centrifuge at 20,000 *g*, 4°C for 30 min and stored at 2°C after being lyophilized.

### Labeling KLH with rhodamine B fluorescence

Ten milligrams of EDC dissolved in 700 μL ultrapure water (pH 6.8) was mixed with 300 μL of 2 mg/mL rhodamine B. After incubation at 0°C for 10 min, the mixture was added with 10 mg KLH (10 mg/mL) and stirred in darkness at room temperature for 12 h. Fluorescently labeled KLH was purified using Microcon centrifugal filter units (50,000 MWCO) from EMD Millipore (EMD Millipore, Billerica, MA, USA) and stored at 2°C after freeze dry.

### Physicochemical property characterization of NPs

Five milligrams of NPs was dispersed in 20 mL ultrapure water (pH 7.0) using a water bath sonicator for 5 min. Each sample was diluted by ten folds using ultrapure water. Particle size (diameter, nm) and surface charge (zeta potential, mV) were measured using a Malvern Nano-ZS zetasizer (Malvern Instruments Ltd, Worcestershire, UK) at room temperature.

### Imaging of NPs using a transmission electrical microscope (TEM)

NPs suspended in ultrapure water (5 mg/mL) were dropped onto a 300-mesh Formvar (Agar Scientific, Essex, UK)-coated copper grid. After 10 min standing, the remaining suspension was carefully removed with wipes, and the samples were negatively stained using fresh 1% phosphotunstic acid for 60 s and washed by ultrapure water twice. The dried samples were imaged on a JEOL JEM 1400 Transmission Electron Microscope (JEOL Ltd., Tokyo, Japan).

### Confocal imaging of LPK NPs

Fluorescent LPK NPs were formed using the above-described methods, except that KLH were labeled with rhodamine B and 0.5 mg of NBD PE was added into existing lipids (DOPC:DSPE-PEG = 16 mg:4 mg). One hundred microliters of NP suspension (1 mg/mL) was placed onto a glass slide and covered with a cover glass (thickness 0.16 to 0.19 mm) from Fisher Scientific (Pittsburgh, PA). The sample was imaged using a Zeiss LSM 510 Laser Scanning Microscope (LSM) (Carl Zeiss, Oberkochen, Germany).

### *In vitro* stability of NPs

Twenty milligrams of NPs was suspended in 20 mL 10% (*v*/*v*) human serum (pH 7.4), 10% (*v*/*v*) FBS, and 10 mM PBS, respectively. The suspensions were constantly mixed on a shaker at room temperature for 9 days. One hundred fifty microliter samples were diluted in 2 mL ultrapure water at different time points, and the particle size was measured by Malvern Nano-ZS zetasizer. The measurements were performed in triplicate at room temperature.

### Determination of KLH content in NPs

KLH in NPs was quantified using a modified method [14]. Briefly, 10 mg of NPs was dissolved in 1 mL of 0.1 M NaOH solution and incubated at 2°C for 12 h. The solution pH was adjusted to 7.0 using 1 M HCl. Two hundred microliters of DOC (0.15, *w*/*v*) was added and the final volume was adjust to 2 mL using ultrapure water. After sitting at room temperature for 15 min, the mixture was added with 200 μL of TCA (80%, *w*/*v*) and incubated for 5 min. Samples were vortexed for 2 min and centrifuged at 5,000 *g* for 20 min at room temperature. Pellets were dissolved in 500 μL of SDS (5%, *w*/*v*) containing 0.01 M NaOH. Following the protocol from the supplier, KLH concentration was determined using Micro BCA Protein Assay Kit (Thermo Fisher Scientific Inc., Waltham, MA, USA).

### *In vitro* release of KLH from NPs in human plasma

Five milligrams of NPs containing rhodamine B-labeled KLH was suspended in 1 mL of 10% (*v*/*v*) human serum (pH 7.4) and incubated in darkness (covered by foil) at 37°C. Samples were centrifuged at 10,000 *g* for 15 min at determined time points. The supernatant (200 μL) was added into a blank 96-well plate (Thermo Fisher Scientific Inc., Waltham, MA, USA) and measured using Synergy HT Multi-Mode Microplate Reader (BioTek Instruments, Inc., Winooski, VT, USA) with excitation at 530 nm and emission at 590 nm. The pellets were resuspended in 1 mL of 10% (*v*/*v*) human serum. Release of KLH at certain time points was calculated by using the following equation: KLH release% = Absorbance at certain time point/Total absorbance × 100.

### Flow cytometry measurement of endocytosis of NPs by DCs

JAWSII (ATCC® CRL-11904™) immature DCs from ATCC were cultured with alpha MEM (80%*v*) including ribonucleosides, deoxyribonucleosides, 4 mM l-glutamine, 1 mM sodium pyruvate and 5 ng/mL murine GM-CSF, and FBS (20%*v*) at 37°C, 5% CO_2_ in 24-well plates (CORNING, Tewksbury, MA, USA). NPs were assembled according to the above-mentioned method, except that KLH was labeled with rhodamine B and 0.5 mg of NBD PE was added to existing lipids. One milligram of NPs suspended in 2 mL complete medium with a final concentration of 0.5 mg/mL was added into each well containing 10^6^ cells and incubated for 1, 2, and 3 h, respectively. After incubation, the medium was immediately removed and cells were washed with ultrapure water for five times. Cells were detached from culture plate using trypsin/EDTA solution and centrifuged at 200 *g* for 10 min, and cell pellets were resuspended in 10 mM PBS (pH 7.4). Cell samples were immediately analyzed using flow cytometer (BD FACSAriaI, BD, Franklin Lakes, NJ, USA).

### LSM imaging of endocytosis of NPs by DCs

Cells were cultured in a four-well chamber slide (Thermo Fisher Scientific Inc., Waltham, MA, USA) using the same method described above. NPs (0.1 mg) suspended in 500 μL complete medium with a final concentration of 0.2 mg/mL were incubated with 10^5^ cells for certain times (1, 2, and 3 h) at 37°C, 5% CO_2_. After incubation, medium was immediately removed and cells were washed with ultrapure water for five times. Freshly prepared 4% (*w*/*v*) paraformaldehyde (500 μL) was added into each well, and cells were fixed for 15 min and washed three times using PBS (10 mM, pH 7.4). Fixed cells were permeabilized using 500 μL of 0.1% (*v*/*v*) Triton™ X-100 for 15 min at room temperature and washed three times using PBS (10 mM, pH 7.4). Cells were stained using 500 μL of freshly diluted 1X HCS CellMask™ Blue Stain for 15 min and washed three times using PBS (10 mM, pH 7.4). Cell samples were covered with a glass cover and sealed by nail polish. Images were acquired using a Zeiss LSM 510 Laser Scanning Microscope (Carl Zeiss, Germany). Each step was carried out in darkness as much as possible to avoid fluorescence quenching.

### Statistical analysis

All experiments were performed in at least triplicate. Results were expressed as mean ± standard deviation. Different treatment groups in stability test were compared by one-way ANOVA following Tukey test using the JMP pro 10 (SAS, Cary, NC, USA). Differences were considered significant at *p* values that were less than or equal to 0.05.

## Results and discussion

### Characterization of PK NPs and LPK NPs

**Figure 1:**
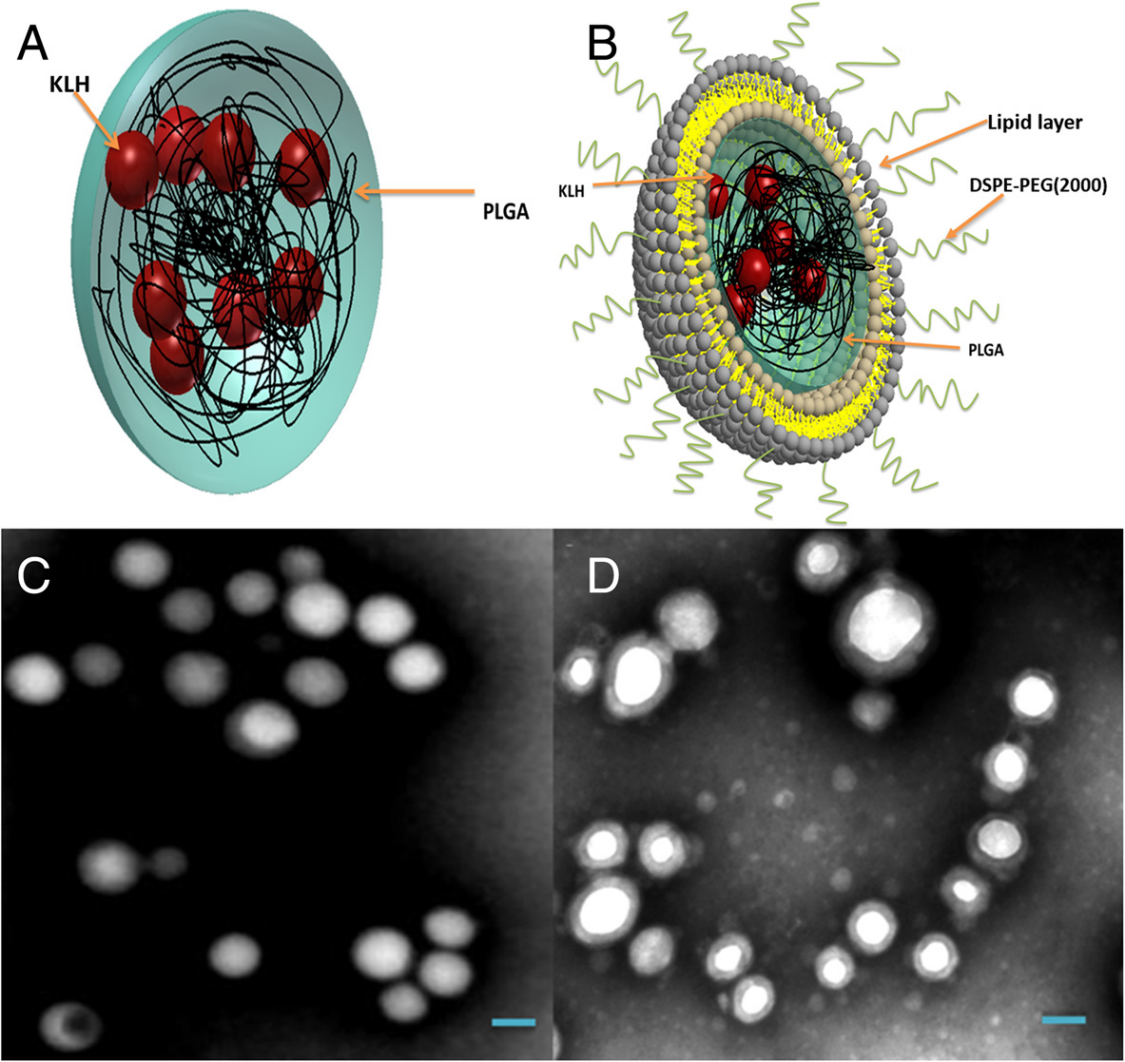
**Schematic illustration and TEM images of the NPs. (A)** Schematic illustration of PK NPs. **(B)** Schematic illustration of LPK NPs. **(C)** TEM image of PK NPs, which highlights the uniform size and spherical shape of PK NPs. **(D)** TEM image of hybrid LPK NPs, which shows the lipid-bilayer-enclosed PK NPs. The scale bars represent 200 nm.

PK NPs (schematically illustrated in Figure 1A) were prepared through double emulsion and evaporation technique, and LPK NPs (schematically illustrated in Figure 1B) were generated from sonication-aided fusion of PK NPs into liposomes. The physicochemical properties, including particle size, polydispersity, surface charge, and antigen content of the NPs, were characterized. In PK NP preparation, 3 mg of KLH was added into 200 mg PLGA during the primary emulsion, and the results indicated that around 75% of the KLH was entrapped inside PLGA. The KLH contents in LPK NPs were slightly less (Table 1), and the decrease is possibly due to the extra weight from the liposome and loss of KLH during LPK NP preparation. Table 1 also shows that PK NPs have a size of 191.0 ± 15.3 nm, while all LPK NPs, ranging from 208 ± 12.0 to 232 ± 34.5 nm, are slightly bigger. Such an increase in size is probably caused by the addition of a lipid layer on the surface of the PLGA NP [15]. Nevertheless, all NPs are well smaller than 500 nm, a size that has been shown to enable the NPs to be efficiently uptaken by DCs for vaccine applications [16]. The low polydispersity value (lower than or equal to 0.240 ± 0.019) for each NP indicates that the size distributions of all NPs are in a very narrow range, reflecting high effectiveness and robustness of the preparation method. The surface charge of LPK NPs expressed as zeta potential is largely dependent on the composition of the lipid layer. PK NPs with carboxyl groups on the surface showed the lowest zeta potential (-9.7 ± 1.1 mV) among all NPs. Compared to PK NPs, LPK^--^ NPs exhibited positively shifted zeta potential, which might be attributed to the shielding effect of DSPE-PEG (2000) and the small amount of amine groups on PEG molecules [17]. The positive zeta potentials of LPK^++^ and LPK^+^ NPs are probably attributed to the positive charges carried by DOTAP. The results from zeta potential measurement demonstrated that the surface charges of hybrid NPs can be flexibly controlled by modulating the lipid composition.

**Table 1:** Components, physicochemical properties, and KLH content of various NPs

**Group**	**Components of NPs (mg)**					**Size (dm. nm)**	**Polydispersity**	**Zeta potential (mV)**	**KLH content (%)**
	**PLGA**	**KLH**	**DOTAP**	**DOPC**	**DSPE-PEG**				
**PK**	200	3	0	0	0	191.0 ± 15.3	0.199 ± 0.012	-9.7 ± 1.1	1.12 ± 0.21
**LPK**^**++**^	200	3	16	0	4	213 ± 38.7	0.231 ± 0.022	13.9 ± 1.3	1.11 ± 0.22
**LPK**^**-**^	200	3	2	14	4	232.4 ± 34.5	0.248 ± 0.018	-3.6 ± 1.4	1.05 ± 0.10
**LPK**^**+**^	200	3	14	2	4	222.6 ± 21.0	0.240 ± 0.019	6.4 ± 1.1	0.92 ± 0.15
**LPK**^**--**^	200	3	0	16	4	208.0 ± 12.0	0.219 ± 0.023	-5.5 ± 0.9	0.84 ± 0.03

Incorporation of long-chain PEG molecules on the surface of NPs is of significant importance as they can not only protect NPs from degradation by enzymes during *in vivo* circulation [18], increasing the stability of NPs and prolonging circulation time [19], but also allow the inclusion of reactive groups in PEG molecules to offer flexible conjugation of various antigens [20]. For targeted delivery purposes, antibodies or affinity ligands against receptors of target cells or tissues may be conjugated to the surface of NPs via PEG chains [21,22].

**Figure 2:**
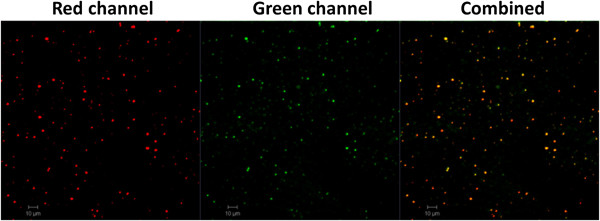
**Confocal images of LPK NPs.** The images illustrate that KLH was labeled with rhodamine B (red) and liposome was labeled with NBD (green), confirming that PK NPs were enclosed by liposome. Scale bars represent 10 μm.

The morphology of NPs was studied using TEM. Consistent with the particle size measured using dynamic light scattering (DLS) (Table 1), both PK NPs (Figure 1C) and LPK NPs (Figure 1D) displayed a highly uniform particle size (around 200 nm) and narrow size distribution. Most of the NPs showed a smooth surface and were of a spherical shape. Compared to PK NPs, there is a gray membrane covering LPK NPs (Figure 1D), demonstrating the successful hybridization of PK NPs and liposomes. The thickness of the membrane is around 20 nm, which is equal to the thickness of a lipid bilayer [15].To further confirm that PK NPs were successfully hybridized with lipids, LPK NPs comprising PK NPs (KLH was labeled with rhodamine B (red color)) and lipid layers (lipids were labeled with nitro-2-1,3-benzoxadiazole (NBD) (green color)) were examined using confocal LSM. Figure 2 shows that the hybrid NPs are marked with both red and green fluorescence, confirming that there is a lipid membrane layer on the surface of PK NPs. It is worth noting that the majority of NPs are double-color labeled, indicating the high efficiency of sonication-induced hybridization of PLGA NPs and liposomes.

### Stability of NPs in PBS, FBS, and human serum

**Figure 3:**
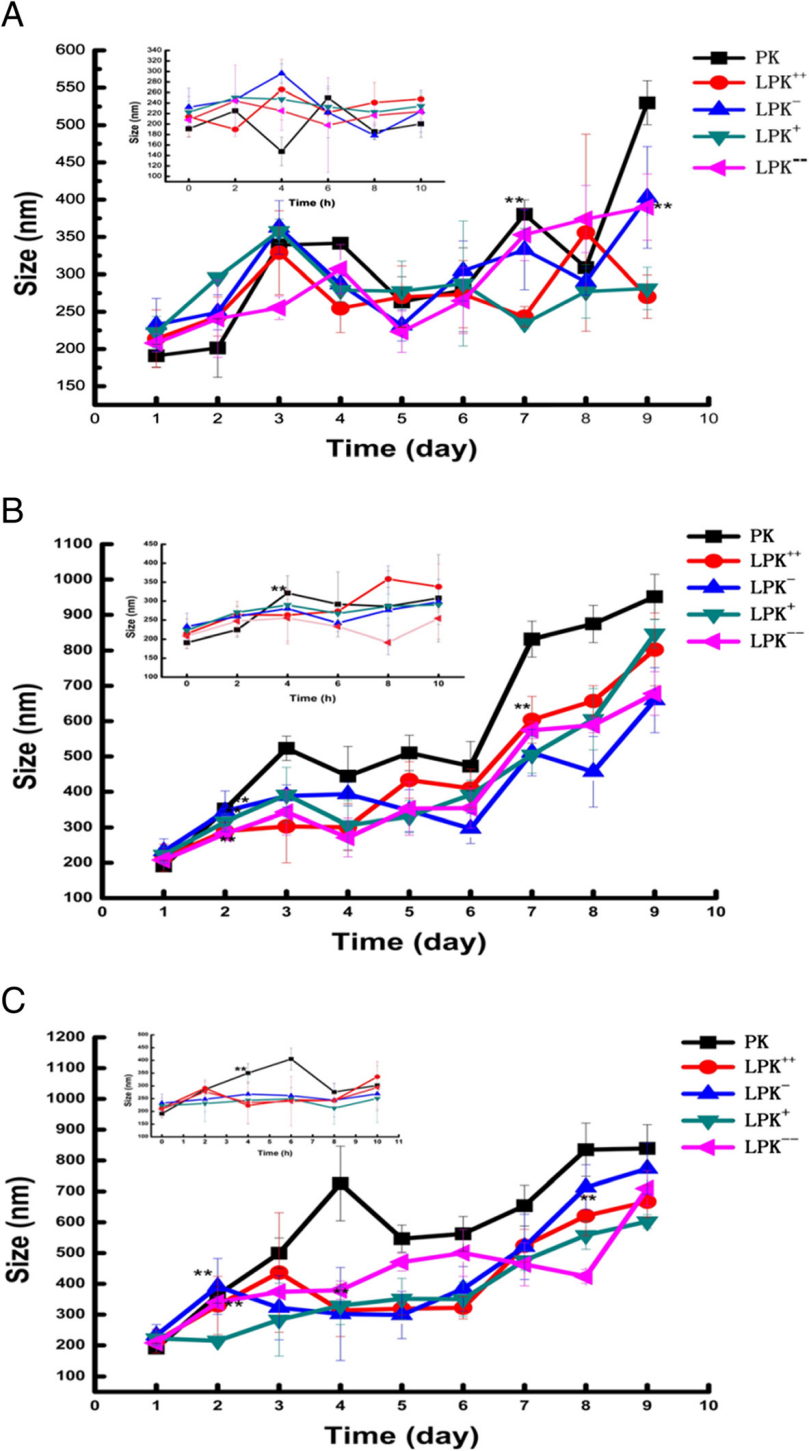
***In vitro*stability of NPs.** Size stability of NPs in various solutions: **(A)** 10 mM PBS, **(B)** 10% (*v*/*v*) FBS, and **(C)** 10% (*v*/*v*) human serum. Sizes of all NPs, except PK NPs, were stable in PBS over 9 days of incubation. LPK NPs demonstrated superior stability compared to PK NPs in the three solutions. In both FBS and human serum, sizes of all NPs increase more quickly compared to that in PBS. The inserts show antigen release from NPs within 10 h of incubation. Double asterisks indicate that the size of NPs at this point was significantly higher compared to that at 0 h (*p* value <0.05).

For vaccines, having a desirable stability could ensure prolonged circulation in blood and sustained induction of immune response. Size stability of NPs in various solutions, (a) 10 mM PBS, (b) 10% (*v*/*v*) FBS, and (c) 10% (*v*/*v*) human serum, was evaluated by DLS (Figure 3). All the NPs, especially LPK NPs, were highly stable during incubation in 10 mM PBS (Figure 3A): no significant size change of LPK NPs was detected over 8 days of test; the size of PK NPs did not increase until day 7. In both FBS (Figure 3B) and human serum (Figure 3C), a marked size change was detected for PK NPs after 4 h of incubation. In contrast, all the LPK NPs stayed stable for at least 2 days in both FBS and human serum. Especially LPK^++^ NPs kept a constant size in FBS for 7 days and in human serum for 8 days. Interestingly, size stability of LPK NPs appears to be related to lipid compositions; NPs with more positive charges exhibited higher stability compared to those with less positive charges. Higher stability of positively charged hybrid NPs may have resulted from a strong electrostatic attraction between cationic lipid layer and anionic PLGA core [22,23].

### *In vitro* release of antigen from NPs

**Figure 4:**
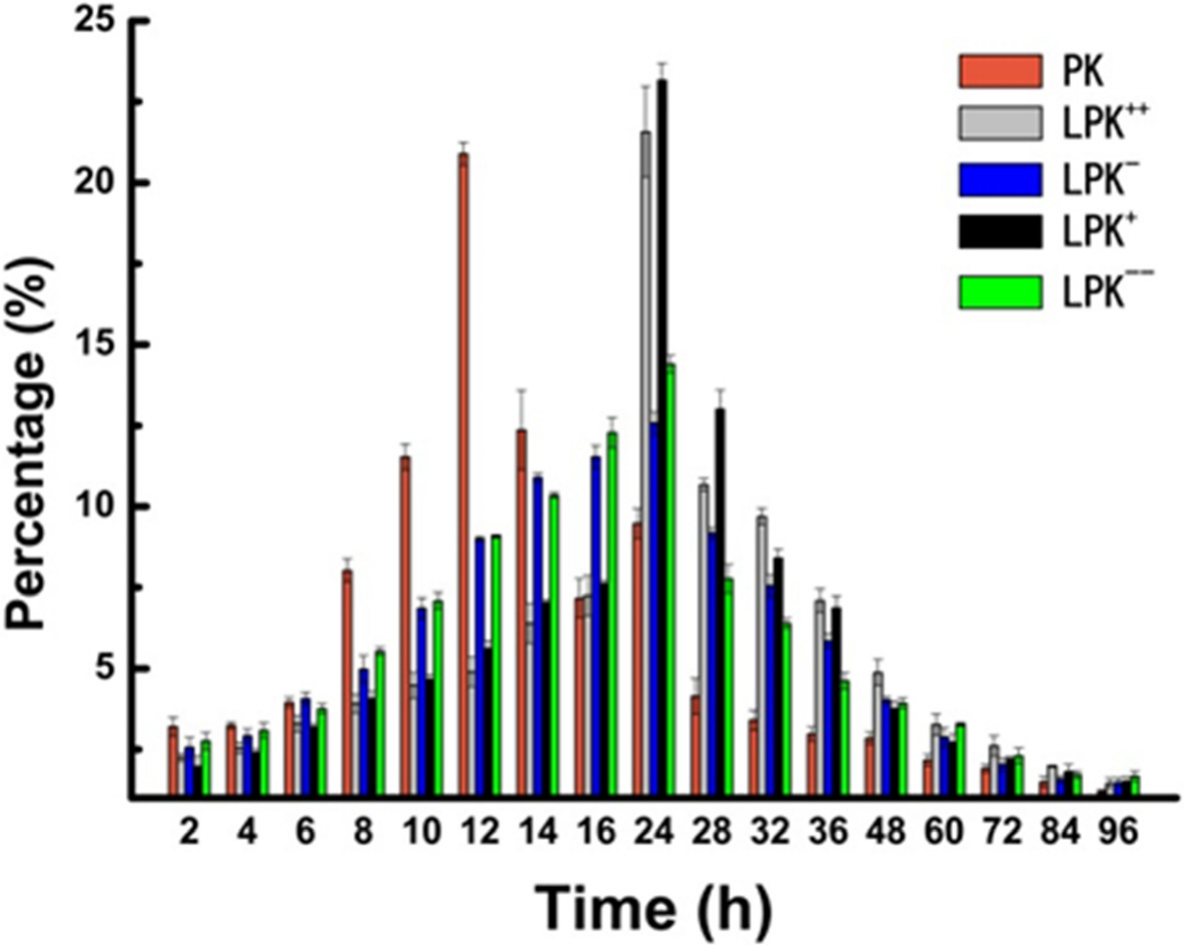
**Release of KLH contained in NPs in 10%human serum (pH 7.4) at 37°C.** All NPs exhibited a prolonged release of KLH. PK NPs showed a burst release of KLH between 8 and 10 h. LPK displayed a delayed release profile, in which the largest percentage release occurred between 16 and 24 h. The extent of release was also dependent on the composition and charge of the NPs.

The evaluation of *in vitro* antigen release from NPs in human serum could simulate the antigen release *in vivo*. In agreement with other reports that a lipid shell could help retain molecules loaded inside PLGA cores [15], in this work, LPK NPs displayed more controlled and delayed release of the payload, KLH. As shown in Figure 4, a burst release was observed between 10 and 12 h for PK NPs, and more than 70% of KLH was released in the first 16 h. In contrast, more than 50% of the KLH was released between 16 and 96 h for LPK NPs, and in particular, only 35% and 37% percent of KLH were released in the first 16 h for LPK^**++**^ and LPK^**+**^ NPs, respectively. The regulated release of KLH in LPK NPs is probably due to the presence of a lipid bilayer that acts as a barrier to reduce KLH diffusion from the PLGA core to the bulk solution and the PEG shield that delays the enzymatic degradation of NPs [24]. Consistent with the results from size stability study, antigen release from NPs with more positive surface charges was slower than the release from NPs with less positive charges. The slower antigen release may be attributed to the tighter association of the lipid layer with the PLGA core, which reduces the diffusion of KLH from NPs into the bulk solution. Delayed antigen release from NPs may reduce loss of antigen during circulation and increase bioavailability of antigen to DCs, thereby enhancing immune response.

### Endocytosis of NPs by DCs

**Figure 5:**
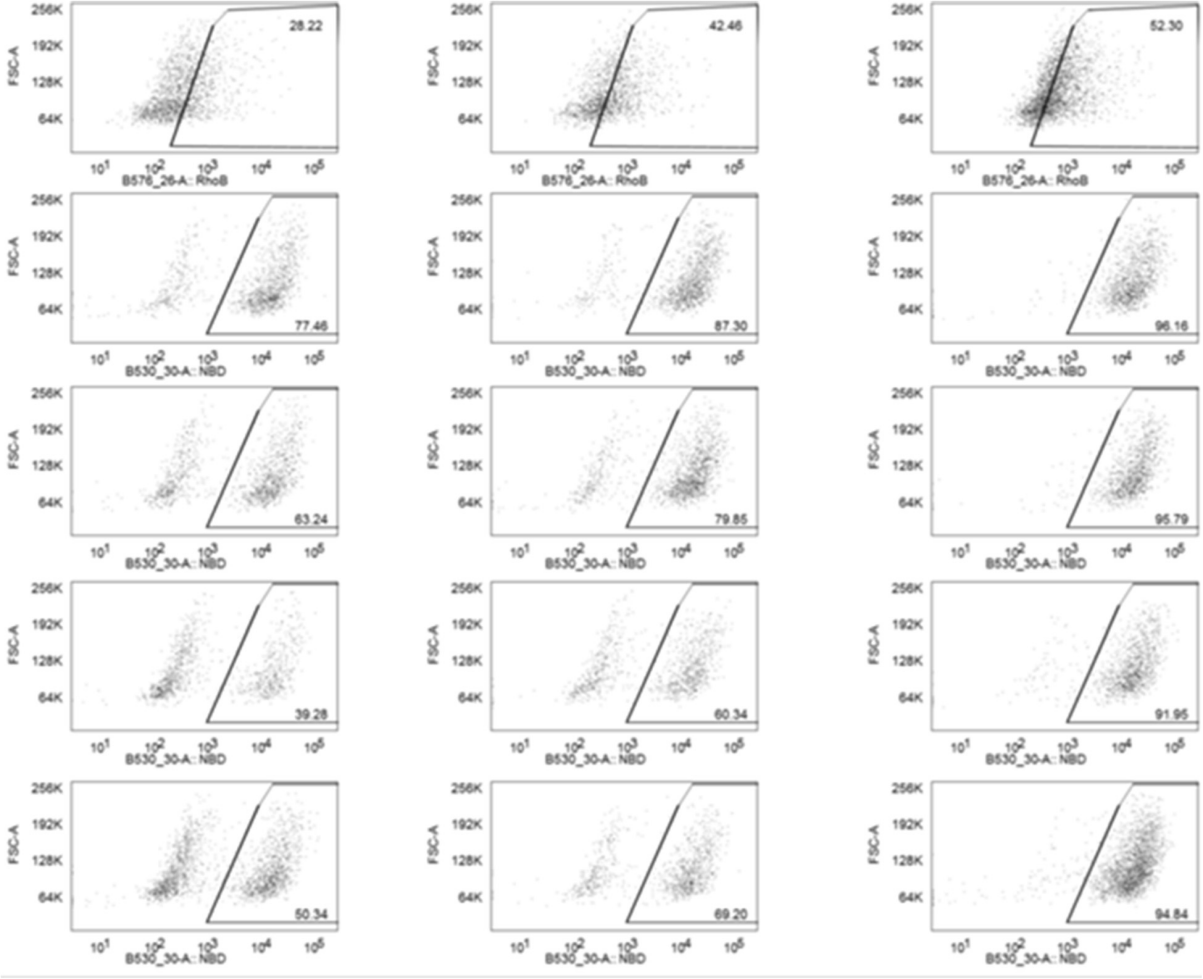
**Flow cytometry measurement of uptake of PK NPs and LPK NPs by JAWSII DCs.** One milligram of NPs was incubated with 10^6^ cells for 1, 2, and 3 h, respectively. As time lapsed, more NPs were ingested by cells. Enhanced uptake of LPK NPs by DCs was observed compared to PK NPs. DCs are more readily to uptake positively charged NPs compared to negatively charged NPs. Most of the cells (>90%) had taken up LPK NPs in 3 h, while only 52% of the cells had taken up PK NPs.

**Figure 6:**
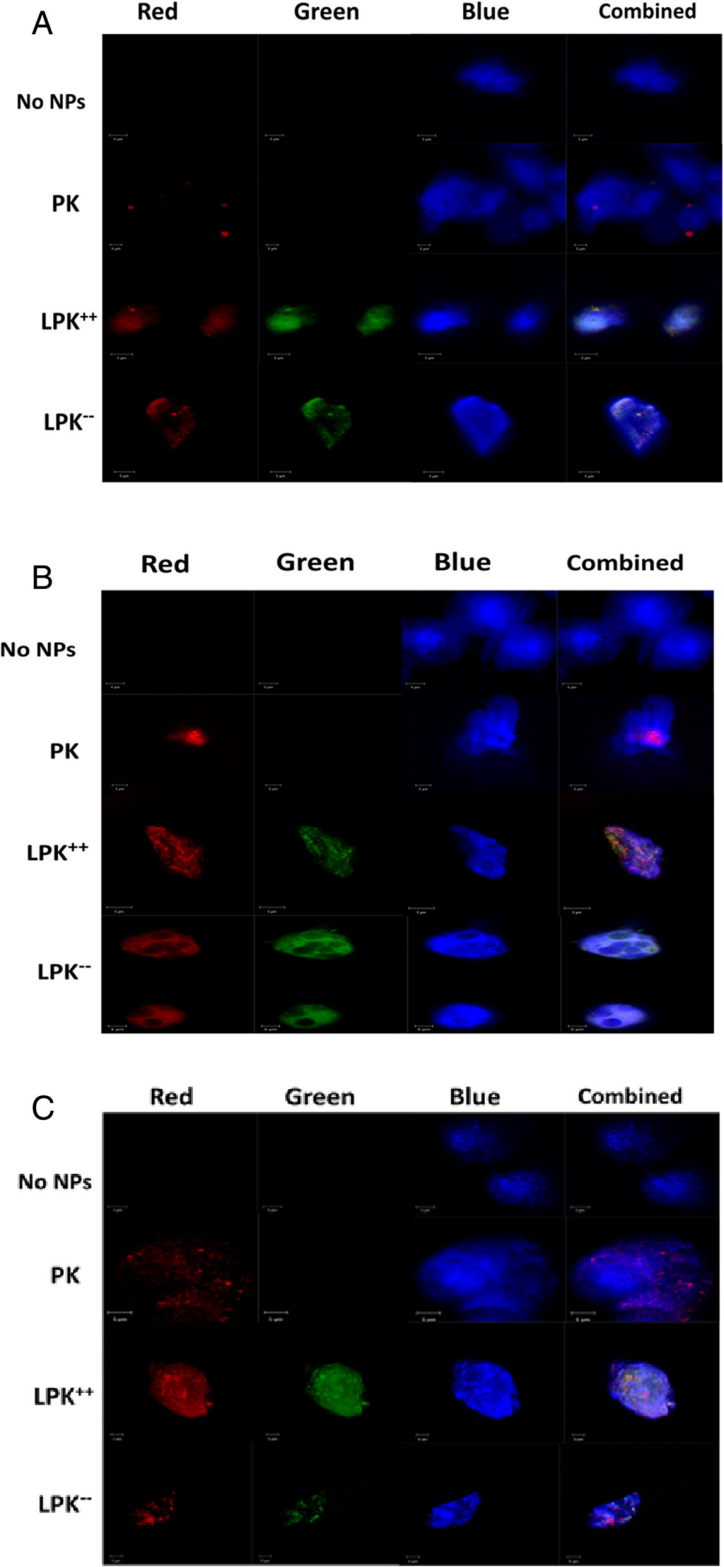
**Confocal images of internalization of PK NPs and LPK NPs by JAWSII DCs.** One hundred thousand cells were incubated with 0.1 mg NPs for 1 h **(A)**, 2 h **(B)**, and 3 h **(C)**, respectively. The incubation concentration was 0.2 mg/mL. Red color is from rhodamine B, which was used to label KLH; green color is from NBD PE, which is a fluorescent lipid used to label the lipid layer; and blue color is from CellMask™ Blue Stain, which was used to label the cell membrane. Both positively charged LPK NPs and negatively charged LPK NPs were internalized more readily by cells than PK NPs. Scale bars represent 5 μm.

DC is the most professional antigen-presenting cell that can initiate and regulate adaptive immune response [25,26]. Higher internalization efficiency of NPs by DCs may lead to more activated T helper cells, resulting in enhanced immune response. Fluorescently marked NPs were added into immature DCs from mouse to study the uptake of NPs by DCs. Results from flow cytometry measurement (Figure 5) showed that higher internalization efficiency was observed in all LPK NPs compared to PK NPs. In the first hour after NP treatment, only 28% of DCs had taken up PK NPs while 77%, 63%, 39%, and 50% of DCs had taken up LPK^++^, LPK^+^, LPK^--^, and LPK^-^ NPs, respectively. After 3 h of incubation, more than 90% of DCs have internalized LPK NPs in all four groups; however, only 52% of DCs have taken up PK NPs. Evidently, surface charge has a great impact on NP uptake. For example, 77% of DCs ingested LPK^++^ NPs in the first hour of incubation, but only 39% for LPK^**--**^ NPs. Faster uptake of NPs by DCs is important because it should reduce the clearance of NPs by reticuloendothelial system (RES), avoid premature degradation by enzymes, and increase the availability of antigens to the immune system. LSM images (Figure 6) also confirmed that LPK NPs had superior uptake efficiency in comparison to PK NPs. In the first hour after NP treatment, only few PK NPs were internalized by DCs; in contrast, both LPK^++^ and LPK^--^ NPs with large quantities were taken up by DCs (Figure 6A). After 2 h, the internalized PK NPs were located in a small area of the cell, while LPK NPs were widely distributed in cells (Figure 6B). Faster uptake of LPK NPs by DCs is probably due to the coating lipid bilayer that could mimic the cell membrane to fuse with the plasma membrane of DCs. Consistent with the results from flow cytometry study, more LPK^**++**^ NPs were ingested by DCs than LPK^**--**^ NPs within the same period of time, suggesting that DCs could more efficiently capture NPs with more positive surface charges. The faster uptake of LPK^++^ NPs may be due to the electrostatic attraction between the positive surface charges on LPK^**++**^ and the negative charges on the plasma membrane of DCs.

## Conclusions

In summary, lipid-PLGA hybrid NPs with variable lipid compositions were constructed. As a potential antigen delivery system, lipid-PLGA NPs exhibited superior quality in comparison to PLGA NPs in terms of stability, antigen release, and particle uptake by DCs. The *in vitro* performance of lipid-PLGA NPs was highly influenced by the composition of the lipid layer, which dictates the surface chemistry of hybrid NPs. Hybrid NPs enveloped by lipids with more positive surface charges demonstrated higher stability, better controlled release of antigen, and more efficient uptake by DCs than particles with less positive surface charges. The results should provide basis for future design of lipid-PLGA hybrid NPs intended for antigen delivery.

## Competing interests

The authors declare that they have no competing interests.

## Authors’ contributions

YH carried out the experiments and drafted the manuscript. ME participated in the design of the experiments. KF participated in the experiments related to dendritic cell culture. CZ conceived the study, participated in its design and coordination, and revised the manuscript. All authors read and approved the final manuscript.
